# Invasive stratified mucin-producing carcinoma of the colorectum: expanding the morphologic spectrum of large bowel cancer

**DOI:** 10.1186/s13000-023-01396-8

**Published:** 2023-10-18

**Authors:** Finn Morgan Auld, Terence N. Moyana

**Affiliations:** Department of Pathology & Laboratory Medicine, University of Ottawa and The Ottawa Hospital, 501 Smyth Road, General Campus, Ottawa, ON K2H 1L6 Canada

**Keywords:** Colorectal adenocarcinoma, Tumor subtypes, Invasive stratified mucin-producing carcinoma

## Abstract

**Background:**

Invasive stratified mucin-producing carcinoma is a recently recognized adenocarcinoma with distinctive features. It was first described in the cervix but similar tumors have since been reported in the penis, anus and prostate. In the gastrointestinal tract, the phenomenon of epithelial stratification has an interesting embryologic morphogenesis. Gastrointestinal mucosa starts off as nascent columnar epithelium that is subsequently patterned to confer regional specific functions. However, in disease states, normal architectural patterning can be disrupted by aberrant differentiation. Given this background and the phenotypic plasticity of neoplastic cells, we were interested in ascertaining whether invasive stratified mucin-producing carcinoma occurs in the colorectum.

**Methods:**

This was a retrospective study of all 584 cases of colorectal carcinoma accessioned at our institution over a 2-year period (January 2021- December 2022). Cases were analyzed to determine which fulfilled the criteria for invasive stratified mucin-producing carcinoma.

**Results:**

There were 9 cases of colorectal invasive stratified mucin-producing carcinoma—one pure form and 8 mixed. They showed the classic colorectal (CK20 + , CDX2 + , CK7-) immunostaining profile but, based on various morphologic criteria, they could be distinguished from conventional adenocarcinoma NOS, mucinous, signet ring cell, medullary, goblet cell and undifferentiated carcinomas. About half the cases were MLH1/PMS2 deficient and BRAF &/or PIK3CA mutated, which aligns with the hypermutated phenotype.

**Conclusions:**

Colorectal invasive stratified mucin-producing carcinoma appears to be a real entity, best recognized in its early stages. It appears to be a high-grade carcinoma. With tumor progression, it evolves into a mucinous adenocarcinoma with a proclivity towards signet ring cells. In summary, the study of this tumor, particularly in its early stages, provides useful clues to further understanding the biology and progression of large bowel cancer. Further studies are required to learn more about this tumor.

## Background

Invasive stratified mucin-producing carcinoma (ISMC) is a newly described adenocarcinoma with distinctive features [1–5]. Morphologically, it is characterized by invasive groups of stratified, immature epithelial cells with peripheral palisading and pushing margins. The tumor cells show at least focal amounts of intracytoplasmic mucin dispersed throughout the entirety of lesional epithelium but without overt gland formation. The tumor was first described in the cervix as a human papilloma virus (HPV)-associated neoplasm. Similar tumors have since been reported in other sites such as the penis and the anus, with or without an association with HPV [6–10]. An analogous phenomenon of epithelial stratification has also been recently described in the non-cribriform variant of prostatic adenocarcinoma and in transition zone adenocarcinomas [11, 12]. In addition, more recently, benign forms of stratified intraepithelial mucinous proliferation of the cervix have been documented without an association with HPV [13].

From a gastrointestinal (GI) perspective, the concept of epithelial stratification has an interesting embryologic morphogenesis. GI mucosa is endoderm-derived and starts off as columnar nascent epithelium that is subsequently patterned to confer regional-specific functions along the cephalocaudal axis [14, 15]. This results in cytodifferentiation into a diversity of epithelial cell types that is mediated by key transcription factors, signaling pathways and epithelial-mesenchymal interactions. In notably 2 sites, the esophagus and anal canal, there is a stratified squamous/columnar epithelium transition zone that is carefully regulated during embryologic development and maintained that way throughout life. However, in disease states, aberrant differentiation from pluripotent stem cells can disrupt the normal architectural patterning of the epithelium e.g. intestinal metaplasia in Barrett's and Paneth cell metaplasia in inflammatory bowel disease [14, 15].

The phenomenon of epithelial stratification is already well-documented in colorectal neoplasia e.g. squamous morules, squamous/adenosquamous carcinoma or various combinations thereof [16–18]. Given this background and the phenotypic plasticity of neoplastic cells, we were interested in ascertaining whether ISMC occurs in the colorectum.

## Methods

### Case selection

Approval for the study was obtained from our institutional Research Ethics Board. Our surgical pathology records were searched for cases of colorectal adenocarcinoma (CRC). The specimens included major resections, local resections (e.g. transanal endoscopic microsurgery and endoscopic mucosal resections) and polypectomies. Most of the resections had a prior endoscopic biopsy that was positive for carcinoma, and for the review, it was paired with the corresponding resection. All cases accessioned at our institution during the 2-year period from January 1, 2021 to December 31, 2022 were retrospectively analyzed. The specimens were fixed, processed and examined according to standard procedures. For cases that had received neoadjuvant treatment, particular attention was paid to therapy-induced changes such as inflammation, fibrosis and acellular mucin as possible morphologic confounding factors; in these cases, the prior diagnostic biopsy helped in determining the original histology. The electronic medical records of the patients were reviewed to exclude the possibility of direct spread/metastasis from some other primary e.g. gastric, breast and lymphoma.

### Histologic assessment

The slides were reviewed to determine whether there were any cases fulfilling the diagnostic criteria of ISMC as stipulated in previous publications [1–9], namely: i) stromal invasive carcinoma composed of solid peripherally-palisaded nests of tumor cells containing variable amounts of intracytoplasmic mucin stratified throughout the entire tumor thickness ii) the tumors were regarded as pure if ≥ 90% of the tumor was ISMC and iii) mixed if the ISMC component was ≥ 10% and < 90%. iv) Tumors with < 10% of the ISMC component were excluded from the study.

### Special stains and immunohistochemistry

To aid in the morphologic assessment of ISMC, every putative case was stained for mucicarmine as well as CK7, CK20, CDX2, synaptophysin, chromogranin, p40, MUC 2, MUC 4, p16 and p53 using the Bond Max staining system. This panel allowed us to be more definitive in our selection criteria. The mucicarmine stain highlighted the intracytoplasmic mucin in the stratified cells. When combined with the CK7, CK20 and CDX-2 immunophenotype, it was helpful in ruling out medullary (aberrant immunoprofile) and undifferentiated carcinomas [19–22]. MUC2 and MUC4 helped in providing quantitative and qualitative aspects to the nature of the mucin—MUC2 is the main constituent of secreted intestinal mucin whereas MUC4 is membrane-associated [23, 24]. The pattern of staining for synaptophysin and chromogranin assisted in ruling out neuroendocrine neoplasms and goblet cell carcinomas. P40 was used to assist in excluding the possibility of squamous differentiation.

### Biomarker testing

Reflex immunohistochemistry testing for DNA mismatch repair protein expression (dMMR) was carried out on all colorectal biopsies that were positive for adenocarcinoma (or resections if there was no biopsy) with no age cutoffs. The standard 4-antibody panel was used (MLH1, MSH2, MSH6 and PMH2. In cases with loss of nuclear expression for MLH1 and PMS2, this was followed by next-generation sequencing (NGS). NGS was also carried out on stage 3 or 4 CRC cases if so requested by the clinicians. Briefly, the tumor was dissected manually without microscopic observation. DNA was analyzed using a targeted NGS assay [Oncomine Comprehensive Assay v3 (OCAv3)]. Given the depth of coverage achieved for this sample, the assay was able to detect DNA variant allele frequencies > 5% for point mutations and small insertions and deletions with a sensitivity > 95%. Genomic regions analyzed include KRAS (exons 2,3,4), NRAS (exons 2,3,4), BRAF (exon 15) and PIK3CA (exons 9,20).

## Results

### Patient demographics and clinicopathologic features

A total of 584 CRC cases were reviewed during the 2-year period. Nine cases met the diagnostic criteria for IMSC with 1 case being pure ISMC while the other 8 were mixed (Table 1). The group was comprised of 5 women and 4 men with a mean age of 68 years (range 44–81 years). Eight tumors were located in the right colon and 1 in the sigmoid colon. Two of the tumors were associated with a pre-existent adenoma. Follow-up ranged from 7 to 32 months (mean 22 months): 5 patients showed no evidence of disease, 3 were alive with disease, and 1 died of disease.

**Table 1:** Clinicopathologic features of the 9 cases of ISMC

	Case 1	Case 2	Case 3	Case 4	Case 5	Case 6	Case 7	Case 8	Case 9
Age, sex	74, F	66, M	56, F	66, F	82, F	77, F	76, M	71,M	44, M
Colorectal site	Ascending colon	Ascending colon	Cecum	Sigmoid colon	Transverse colon	Hepatic flexure	Cecum	Ascending colon	Hepatic flexure
Tumor size	2.1 × 1.5x0.5	3.0 × 2.5x0.8	5.5 × 5.0x1.2	5.0 × 3.0x2.5	15.4 × 10.0x3.1	4.5 × 2.6x1.4	3.6 × 3.5x2.5	5.1 × 4.7x3.2	6.0 × 5.0x2.7
% of ISMC	90%	60%	30%	20%	20%	20%	15%	15%	10%
Pre-existing lesion	TA	None	TVA	None	None	None	None	None	None
LVI	Present	Present	Present	Present	Present	Present	Present	Present	Present
PNI	Absent	Absent	Absent	Present	Absent	Absent	Present	Absent	Present
Tumor stage	T1N0M0	T3N1bM0	T3N1aM0	T4aN2bM0	T4aN2aM0	T3N2bM0	T4bN2bM1a	T4bN2aM1	T4aN2bM0
Appendix	Present; normal	Present; normal	Present; normal	Present; normal	Present; normal	Appendix with SSP	Remote appendectomy	Remote appendectomy	Present; normal
MLH1	Loss	Loss	Loss	Intact	Loss	Loss	Intact	Intact	Intact
MSH2	Intact	Intact	Intact	Intact	Intact	Intact	Intact	Intact	Intact
MSH6	Intact	Intact	Intact	Intact	Intact	Intact	Intact	Intact	Intact
PMS2	Loss	Loss	Loss	Intact	Loss	Loss	Intact	Intact	Intact
KRAS	No mutation	No mutation	No mutation	No mutation	No mutation	No mutation			No mutation
NRAS	No mutation	No mutation	No mutation	No mutation	No mutation	No mutation			No mutation
BRAF	BRAF V600E (c.1799 T > A) mutation	BRAF V600E (c.1799 T > A) mutation	No mutation	No mutation	BRAF V600E (c.1799 T > A) mutation	BRAF V600E (c.1799 T > A) mutation			No mutation
PIK3CA	PIK3CA mutation (p.Glu81Lys)	PIK3CA mutation (p.Glu81Lys	No mutation	No mutation	PIK3CA mutation (p.Glu54Lys)	No mutation			PIK3CA mutation (p.Glu81Lys)
Post-surgical treatment & Follow up	No chemotherapy. 12 months. Alive with no disease	Chemotherapy. 27 months. Alive with no disease	Chemotherapy. 30 months. Alive with no disease	Chemotherapy. 22 months. Alive with no disease	Declined chemotherapy. 18 months. Alive with disease	Chemotherapy. 21 months. Alive with disease	Chemotherapy. 27 months. Alive with disease	Chemotherapy. 7 months. Died of disease	Chemotherapy. 32 months. Alive with no disease

### Morphologic evaluation

**Fig. 1:**
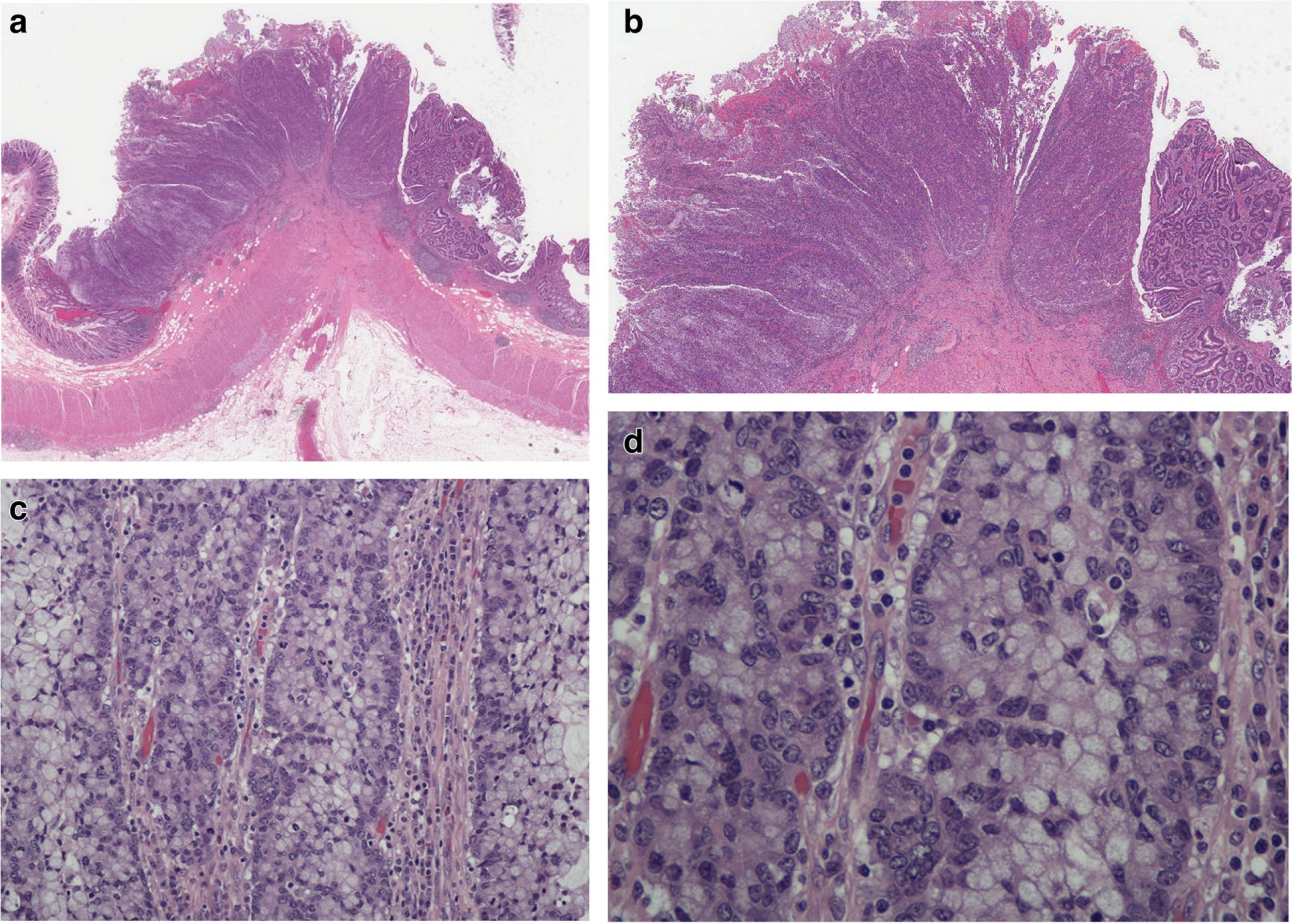
**a** Photomicrograph of case 1 (pure ISMC) showing the tumor in the centre (stage pT1), tubular adenoma on right and normal mucosa on either side. (H&E; original magnification × 0.6). **b** Low power showing the lobulated pushing margin of the tumor to the left and centre. (H&E; original magnification × 2). **c** The tumor showing a trabecular pattern with peripheral palisading. There are stratified epithelial cells with variable amounts of intracytoplasmic mucin. The stroma shows a lymphoplasmacytic infiltrate (H&E; original magnification × 20). **d** More detailed cytologic features of the trabeculae. The peripheral cells have round vesicular nuclei and amphophilic cytoplasm (mucin-poor). More mucin-containing cells centrally. (H&E; original magnification × 40)

**Fig. 2:**
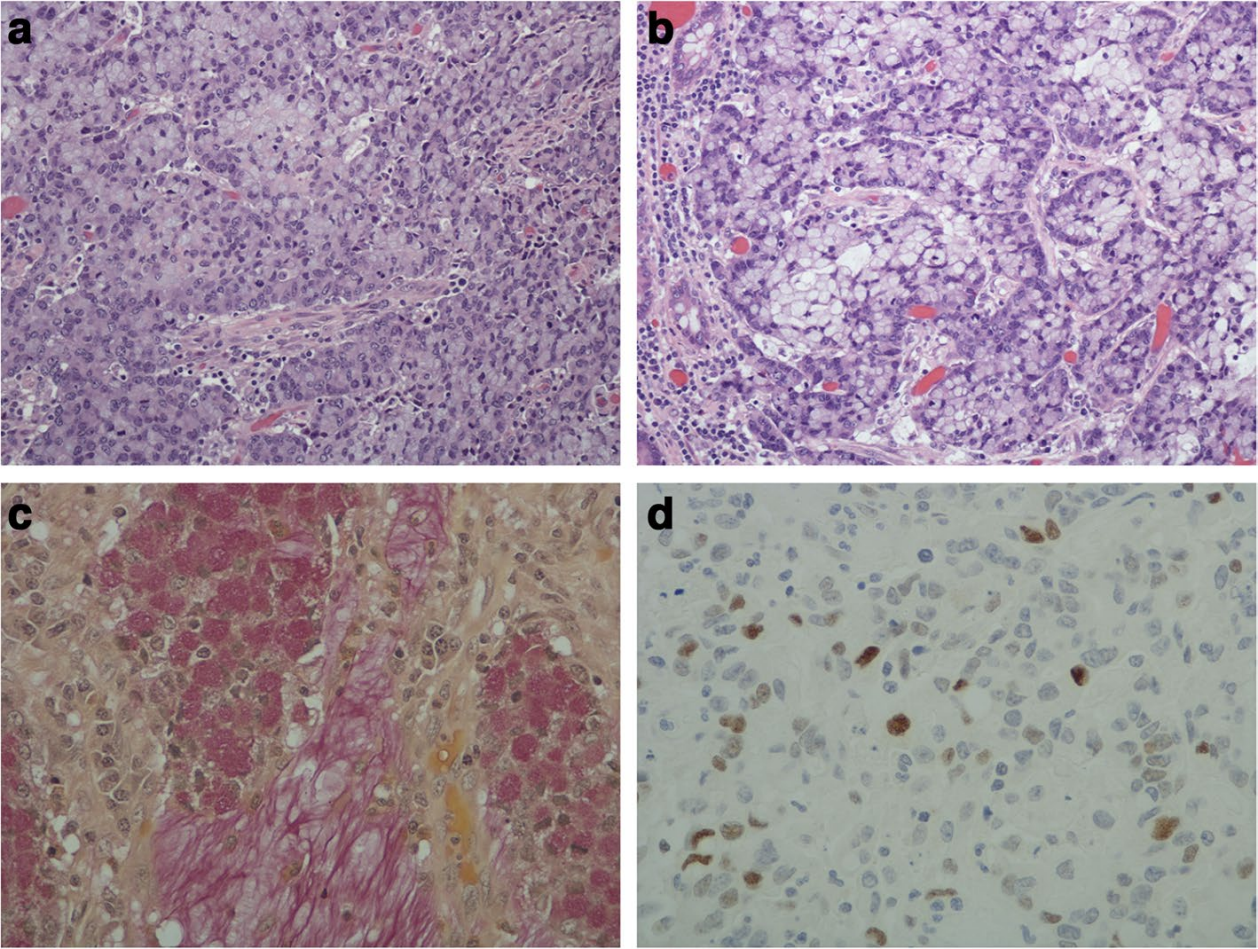
**a** The stratified cells show an anastomosing arborizing pattern that is rimmed by the primitive-looking cells. The stratified mucin-containing cells have clear or slate grey cytoplasm (H&E; original magnification × 20). **b** High-power view showing stratified mucin containing cells with an interdigitating pattern. A few benign colonic mucosal glands are seen on the left. Tumor budding is on the low end of the spectrum. (H&E; original magnification × 40). **c** This is a mucin-rich area with a central focus of cell breakdown and mucin extravasation. Such foci mark the beginning of the mucin component. (Mucicarmine; original magnification × 40). **d** Immunohistochemistry for p53 showing a wild-type pattern in the pure ISMC. (Original magnification × 40)

The one pure ISMC (case 1) was of particular interest and will be described in considerable detail (Figs. 1 & 2). Grossly, it was located in the ascending colon and measured 2.1 by 1.6 cm to a depth of 0.5 cm. It was a relatively early adenocarcinoma (pT1) arising from a pre-existent conventional tubular adenoma (Fig. 1a). The tumor was characterized by well-demarcated, solid, trabecular or insular groups of tumor cells with pushing margins rounded contours (Fig. 1b). The periphery of the cell groups was rimmed by immature-looking-cells with round vesicular nuclei and amphophilic or somewhat clear cytoplasm; these were the relatively mucin-poor areas of the tumor (Fig. 1c). The more central areas tended to have larger amounts of intracytoplasmic mucin (Fig. 1d). Tiny foci of mucin extravasation and early dyscohesion of the tumor cells could be seen. In areas, the tumor had an anastomosing pattern (Fig. 2a) but still with a well-demarcated pushing front and low tumor budding (Fig. 2b). The mucicarmine stain highlighted the variable amounts of intracytoplasmic mucin as well as extravasated mucin (Fig. 2c). The MUC2 showed the mucin in an analogous manner to mucicarmine but MUC4 was patchy. Two tumors showed overexpression for p53 and the rest had variable (wild type) staining (Fig. 2d); none were completely negative (null phenotype).

**Fig. 3:**
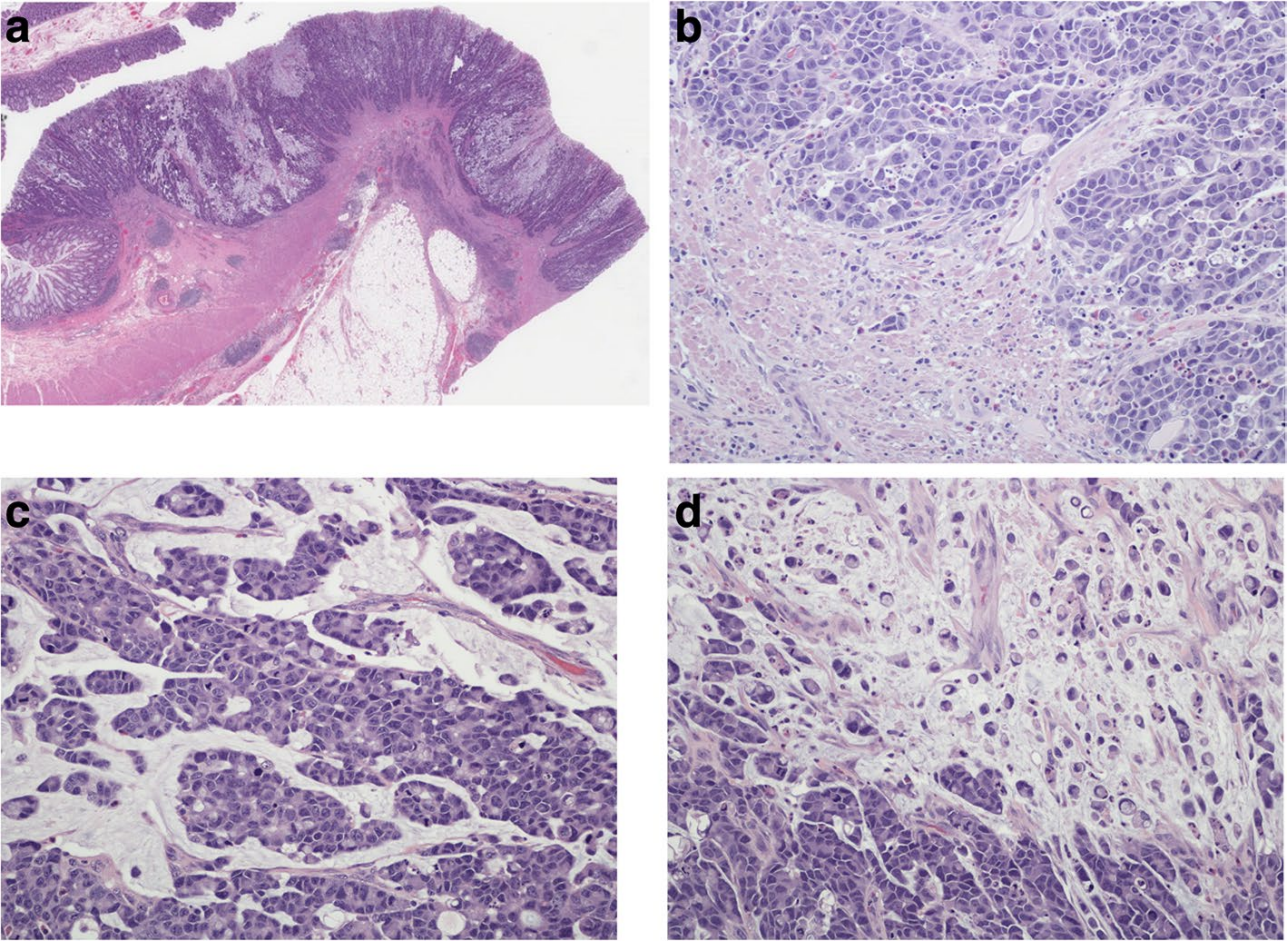
**a** Case 2 (mixed type of ISMC) showing a similar configuration to case 1 i.e. slightly exophytic tumor with a broad undulating base. It was mostly contained by the muscularis propria but a few small foci just managed to push through (stage pT3). (H&E; original magnification × 0.4). **b** Case 2 highlighting the invasive front of the tumor. It still has a pushing margin but a few groups of tumor cells are breaking off and beginning to infiltrate the underlying muscularis propria. Note the small focus of lymphovascular invasion in the central part of the muscle wall. (H&E; original magnification × 20). **c** The stratified cells are showing architectural complexity and variegation. (H&E; original magnification × 20). **d** Field showing a preponderance of signet ring cells in a mucinous background. (H&E; original magnification × 20)

Of the mixed tumors, case 2 (Fig. 3) had the largest component of ISMC (60%). It's configuration was similar to case 1 in that it was slightly exophytic with a broad undulating base (Fig. 3a). The tumor was mostly contained by the muscularis propria but a few small nubbins had broken through the muscle (pT3) (Fig. 3b). The tumor showed more architectural complexity (compared to case 1) in that there was more variation in the size and shape of the cell groups. The combination of groups of stratified immature and mucin-containing cells, mitotic figures and apoptotic bodies in a mucinous background provided quite a variegated checkerboard type picture (Fig. 3c). In other areas, this gave way to a predominance of signet ring cells, singly or in small clusters, in a mucinous background (Fig. 3d). This case showed over-expression of p53 (strong positivity in > 75% of the tumor cells) on immunohistochemistry. The other 7 cases had a smaller ISMC component ranging from 10 to 30% with the main component being mucin pools and signet ring cells. So by the generally accepted definition (> 50% mucinous component), they would be classified as mucinous adenocarcinomas. Overall, based on their proclivity for mucinous and signet ring cell differentiation and/or metastatic behavior, all the 9 cases (pure and mixed ISMCs) in this study appear to be high-grade carcinomas.

All the tumors were positive for CK20 and CDX-2 consistent with the conventional CRC immunophenotype [19–22]. They were negative for CK7 except for a few cases that showed small scattered foci of positivity. None of the tumors showed sizable areas of positivity for neuroendocrine differentiation as determined by synaptophysin and chromogranin. None of the tumors showed block-like positivity for p16. By routine histology, there were no areas of squamous differentiation in any of the tumors; p40 immunohistochemistry was negative even in the relatively mucin-poor peripheral areas. None of the tumors showed goblet cell nests, Paneth-like cells and neuroendocrine cells as encountered in goblet cell carcinoma. From a biomarker perspective, 5 of the tumors (5/9) showed loss of nuclear expression for MLH1 and PMS2 (Table 1). Of these, 4 (4/7) had a BRAF mutation. Four of 7 cases (4/7) a PIK3CA mutation. No tumors (0/7) had KRAS or NRAS mutations.

## Discussion

It is common knowledge that epithelial-lined organs such as the GI, respiratory and the genitourinary systems can develop both adenocarcinomas and squamous cell carcinomas or combinations thereof. One of the hallmarks of squamous cell carcinoma is epithelial stratification. What is not as well-known is the phenomenon of epithelial stratification with intracytoplasmic mucin as has recently been described in the cervix, anus, penis and prostate [1–9]. The current study shows that this phenomenon can also occur in the colorectum. It is different from squamous differentiation in that [16–19] it is a cohesive group of stratified epithelial cells with variable amounts of intracytoplasmic mucin dispersed through the entirety of the lesion but without overt gland formation.

Colorectal ISMC differs from the well and moderately differentiated (low-grade) adenocarcinomas (NOS) in that there are no well-formed conventional glands found in this tumor. With poorly differentiated conventional adenocarcinomas (NOS), the glands are still present though they are highly irregular and more difficult to discern compared to low-grade tumors. Furthermore, these tumors are often quite desmoplastic with irregular infiltrative margins/high tumor-budding scores [22, 25–27], and lack the trans-lesional stratified pattern with peripheral palisading of ISMCs [1–9].

**Fig. 4:**
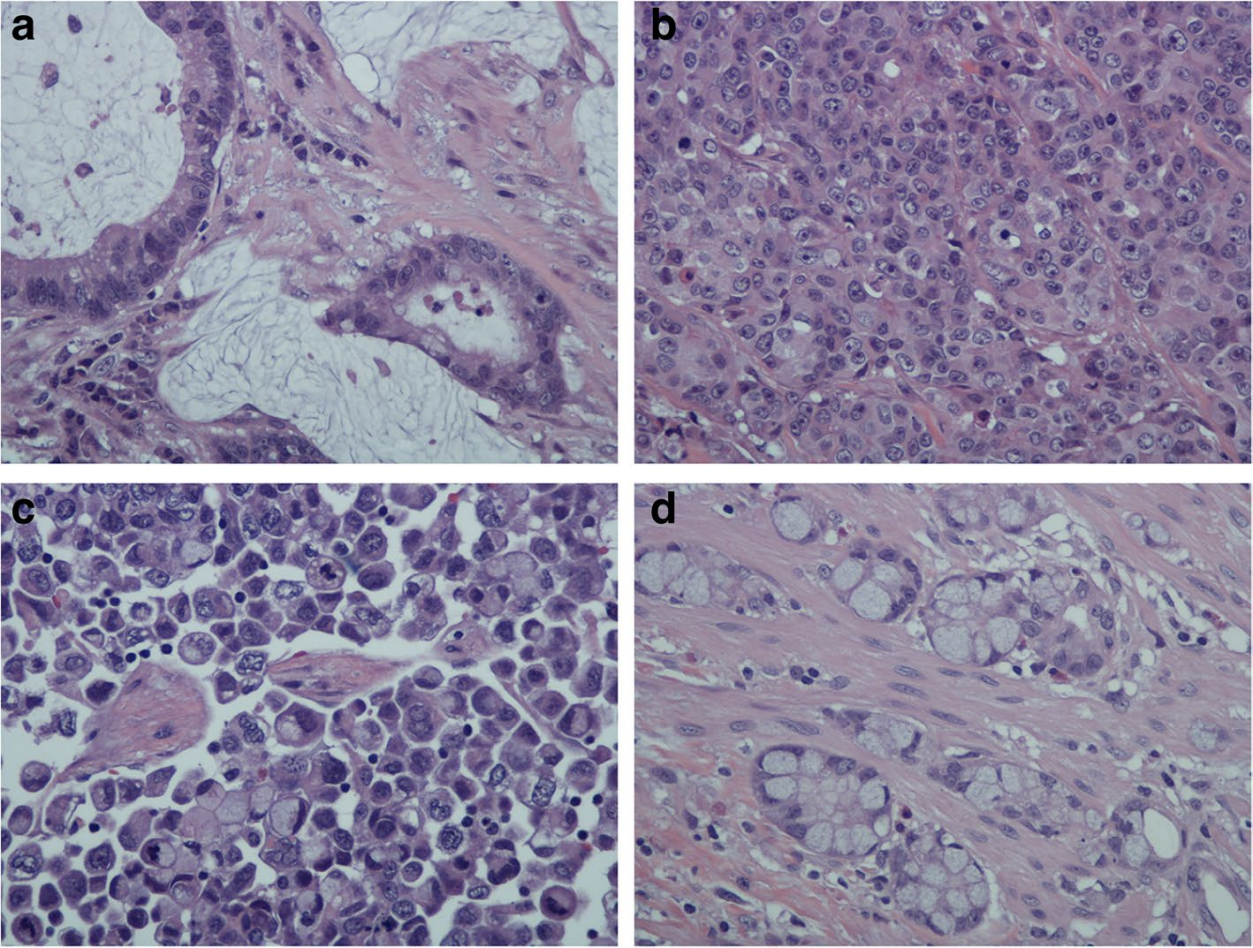
**a** Mucinous adenocarcinoma showing a few profiles of conventional malignant colonic acini associated with pools of extracellular mucin. (H&E; original magnification × 40). **b** Medullary carcinoma showing groups of polygonal cells with vesicular nuclei, prominent nucleoli and abundant cytoplasm arranged in a diffuse or nesting pattern. (H&E; original magnification × 40). **c** Signet ring cell carcinoma showing a predominance of dyscohesive cells with prominent intracytoplasmic mucin and a crescentic nucleus that is displaced to one side. (H&E; original magnification × 40). **d** Goblet cell carcinoma (grade 1) composed of small well-defined nests of goblet-like cells. Immunohistochemistry showed scattered neuroendocrine cells within the goblet cell clusters. Other areas showed Paneth cell differentiation. (H&E; original magnification × 40)

Colorectal ISMC is different from the mucinous adenocarcinoma in its histologic architecture and constituent cells. Mucinous adenocarcinomas generally have conventional glands (Fig. 4a) admixed with (extracellular) mucinous pools (> 50% of the tumor) though there can be variable numbers of signet ring cells (intracellular mucin) [22, 25–27].

ISMC also has to be distinguished from medullary carcinoma [20–22, 25–27] which is largely composed of polygonal cells with vesicular nuclei, prominent nucleoli, and abundant cytoplasm (Fig. 4b). The tumor cells are mostly arranged in sheets, nests or trabeculae with at the most very few isolated glands or mucin. The immunohistochemical profile of these tumors is aberrant (CK7 + /CK20-/CDX2-), thus differing from ISMCs which have a typical CRC immunoprofile (CK20 + /CDX2 + /CK7-). Medullary carcinoma is an important category because, despite its seemingly undifferentiated appearance, it has less frequent lymph node metastasis and a relatively favorable prognosis [20–22, 25–27]. In contrast, 8 of 9 CRC ISMCs in this limited series had lymph node metastases.

Unlike CRC ISMC, undifferentiated carcinomas show no glandular structures or any other features that are indicative of definite differentiation [21, 22, 25, 27]. It is possible that some of the cases originally designated as undifferentiated CRCs may in fact represent medullary carcinoma [20–23, 27].

Another important differential diagnosis is signet ring cell carcinoma (Fig. 4c) or discohesive carcinoma which is mostly seen in the stomach [28–31]. This gastric adenocarcinoma, even in its early stages, appears to begin de novo as signet ring cell carcinoma [29–31]. In fact, since its early stages can mimic mucin neck cells, recent genetic studies and follow up have helped to better recognize and define this entity. In contrast, it has been noted that the origin of colorectal signet ring cell carcinoma is quite confounding with the exception of those tumors that clearly have an origin in the more conventional mucinous adenocarcinomas associated with an overlying adenoma [27]. Tumors meeting the strict definition of CRC signet ring cell carcinoma (> 50% of isolated tumor cells having prominent intracytoplasmic mucin) are rare [27, 32]. They tend to be fairly homogeneous with little if any glandular formation and present at advanced stages with a rapid downward course. In the case of CRC ISMC, the signet ring cells appear to be a product of tumor progression i.e. they are seen admixed with mucinous pools and other cohesive irregularly shaped tumor cell clusters rather than the relatively more homogeneous diffusely infiltrative individual signet ring cells seen in the stomach or colorectum [27–32].

ISMC should also to be distinguished from goblet cell carcinoma (Fig. 4d). This is a tumor of appendiceal origin that characteristically shows discrete tubules or clusters of goblet-like mucinous cells admixed with variable numbers of neuroendocrine cells and Paneth-like cells [33–35]. With progression, there is an increase in the number of signet ring cells, mucinous pools and architectural complexity. Indeed, the recently proposed idea of grading goblet cell carcinomas was meant to address this phenomenon [33–35]. Colorectal ISMC, however, differs from goblet cell carcinoma in that it is neither located in the appendix nor does it demonstrate the amphicrine composition that includes goblet cell nests, neuroendocrine cells and Paneth cells.

All in all, our findings show that CRCs with the histology of ISMC do exist. The study suggests that, with tumor progression, CRC ISMCs develop a more prominent mucinous and signet ring cell component in a manner similar to the increase in complexity and histologic grade that occurs with goblet cell carcinomas [29–31] and other tumors. The importance of this entity is that it highlights the phenotypic plasticity of colorectal progenitor cells, and provides another pathway for the genesis of signet ring cells besides conventional mucinous CRC adenocarcinoma [27]. The experience with these tumors in other organs is still limited, but various authors suggest that it is an aggressive neoplasm [1–5], which is in accord with our study. Further investigations are warranted to learn more about this tumor, focusing on the early lesions that are easier to recognize before tumor progression/dedifferentiation efface the prototypical morphology. In this way, ISMC could be enlisted together with other CRC morphologic subtypes such as micropapillary, adenoma-like, serrated, mucinous, poorly cohesive, signet ring cell, medullary, adenosquamous and sarcomatoid carcinoma [21, 22, 25–27]. Ultimately, all these histologic patterns are a manifestation of the phenotypic plasticity of the progenitor cells. The resultant diversity of carcinoma types does, however, have clinicopathologic and prognostic utility. At the same time, it is increasingly being recognized that there is value to combining the histologic classification with the clinical profile and molecular signatures. For CRC ISMCs, to the extent that they tend to be right-sided, mucinous, lymphoplasmacytic, MLH1/PMS2 deficient with BRAF mutations, they seem to be aligned with the hypermutated molecular phenotype [36–38]. Future studies, both prospective and retrospective, are required to better characterize these tumors.

## Conclusions

Colorectal invasive stratified mucin-producing carcinoma appears to be a real entity, best recognized in its early stages. It appears to be a high-grade carcinoma. With tumor progression, it evolves into a mucinous adenocarcinoma with a proclivity towards signet ring cells. In summary, the study of this tumor, particularly in its early stages, provides useful clues to further understanding the biology and progression of large bowel cancer. Further studies are required to learn more about this tumor.

## Data Availability

The datasets used and/or analysed during the current study are available from the corresponding author on reasonable request.

## Abbreviations

CRC: Colorectal adenocarcinoma
GI: Gastrointestinal
HPV: Human papilloma virus
ISMC: Invasive stratified mucin-producing carcinoma
LVI: Lymphovascular invasion
NOS: Not otherwise specified
PNI: Perineural invasion
